# SIK2 Improving Mitochondrial Autophagy Restriction Induced by Cerebral Ischemia-Reperfusion in Rats

**DOI:** 10.3389/fphar.2022.683898

**Published:** 2022-05-02

**Authors:** Ran Zhang, Yun Liu, Wenhua Zhong, Zebo Hu, Chao Wu, Mengyao Ma, Yi Zhang, Xiangyun He, Lin Wang, Shu Li, Yun Hong

**Affiliations:** ^1^ Department of Pathophysiology, Wannan Medical College, Wuhu, China; ^2^ Department of Clinical, Wannan Medical College, Wuhu, China; ^3^ Department of Public Health, Wannan Medical College, Wuhu, China; ^4^ Department of Ultrasonic Medicine, Yijishan Hospital, Wannan Medical College, Wuhu, China

**Keywords:** SIK2, MCAO, energy metabolism, mitochondrial autophagy, cerebral ischemia-reperfusion

## Abstract

Previous studies have shown that Salt-induced kinase-2(SIK2) is involved in the regulation of various energy-metabolism-related reactions, and it also can regulate angiogenesis after cerebral ischemia-reperfusion. However, it is unclear whether SIK2 can regulate energy metabolism in cerebral ischemia-reperfusion injury. As mitochondria plays an important role in energy metabolism, whether SIK2 regulates energy metabolism through affecting mitochondrial changes is also worth to be explored. In this study, rats were treated with adeno-associated virus-SIK2-Green fluorescent protein (AAV-SIK2-GFP) for the overexpression of SIK2 before middle cerebral artery occlusion (MCAO). We found that SIK2 overexpression could alleviate the neuronal damage, reduce the area of cerebral infarction, and increase the adenosine triphosphate (ATP) content, which could promote the expression of phosphorylated-mammalian target of rapamycin-1 (p-mTORC1), hypoxia-inducible factor-1α (HIF-1α), phosphatase and tensin homologue-induced putative kinase 1 (PINK1) and E3 ubiquitinligating enzyme (Parkin). Transmission electron microscopy revealed that SIK2 overexpression enhanced mitochondrial autophagy. It is concluded that SIK2 can ameliorate neuronal injury and promote the energy metabolism by regulating the mTOR pathway during cerebral ischemia-reperfusion, and this process is related to mitochondrial autophagy.

## Introduction

Ischemic stroke accounts for 80–85% of the cerebrovascular diseases, and it has a high disability and mortality rate, which seriously affects the quality of human life. The reconstruction of blood flow is the most effective method to treat cerebral ischemia. However, in some cases, restoring blood aggravated the development of the disease, which is called Cerebral Ischemia-Reperfusion Injury (CIRI) (Eltzschig and Eckle, 2011). In recent years, some studies have confirmed that the damage involves energy metabolism disorder, autophagy, oxidative stress and so on. Mitochondria are the energy factory of the body and the mitochondrial respiratory chain is the main source of reactive oxygen species (ROS) (Tang et al., 2016; Yang et al., 2018). Therefore, once the mitochondrial structures and functions are destroyed, the energy metabolism will be out of balance, and if the damaged mitochondria can’t be removed in time, excessive ROS will be produced which will eventually cause irreversible cell damages. Severely damaged mitochondria will induce the cell death and many diseases including cerebral ischemia will be aggravated (Bhat et al., 2015). Hence maintaining the mitochondrial integrity is important to resist cerebral ischemia-reperfusion injury.

According to different degradation ways of substrates entering lysosomes, autophagy can be divided into three categories: macroauto-phagy, microautophagy and chaperone-mediated autophagy (CMA). Macroauto-phagy is what we commonly called autophagy and is the most easily induced, which refers to a membrane derived from endoplasmic reticulum wraps around the biodegradable materials to form autophagosomes, fused with lysosomes and degrading their contents (Parzych and Klionsky, 2014). Mitochondrial autophagy is a selective macroauto-phagy, which can remove dysfunctional mitochondria (Liu et al., 2017). The mitophagy will be induced by the starvation (nutritional deficiency) or stress damage (Bravo-San Pedro et al., 2017; Pei et al., 2019), then the damaged mitochondria will be wrapped in the double membrane which are shed from the non-ribosome attachment zone of the rough endoplasmic reticulum to form autophagosomes, which are fused with lysosomes to form autophagolysosomes to degraded the mitochondria content (Yorimitsu and Klionsky, 2005; Wang et al., 2015). Some studies have shown that mitochondrial autophagy is also involved in the regulation of cerebral ischemia-reperfusion injury, but the role it plays remains unclear (Bhat et al., 2015; Flannery and Trushina, 2019). These experiments prove that some interventions play a protective role and can alleviate the cerebral ischemia-reperfusion injury in rats and the OGD/R injury in neurons by promoting mitochondrial autophagy (Sun et al., 2018; He et al., 2019). However, in other experiments, over-activation of mitochondrial autophagy causes delayed cell death and aggravates cerebral ischemia reperfusion injury (Shi et al., 2014; Cui et al., 2020).

Salt-induced kinase (SIK) is a serine/threonine protein kinase, and SIK2 may have similar functions of the adenosine monophosphate-activated protein kinase (AMPK) as a member of its family (Lizcano et al., 2004; Zhao et al., 2020). Studies have found that SIK2 plays an important role in many aspects such as energy metabolism, cell metabolism and tumor. These experiments prove that SIK2 inhibits the anabolism of 3T3-L1 adipocytes (Du et al., 2008). In recent years, other studies have found that SIK2 is located in the centrosome and plays a key role in mitosis initiation, which can affect the sensitivity of ovarian cancer to paclitaxel (Gao et al., 2020). In addition, a report reveals that SIK2 plays an important role in autophagosome maturation and autophagy process, providing a new evidence for the regulatory role of SIK2 in cell nutrition and energy metabolism (Dai et al., 2021). But there are few studies on whether SIK2 also plays a role in regulating energy metabolism in neurons. In this study, we proved that SIK2 could promote the level of energy metabolism and mitochondrial autophagy-related proteins after cerebral ischemia-reperfusion in rats, and increase the level of ATP in brain tissue, which indicates that it plays a protective role in brain injury. We hope this study can provide a new perspective for the clinical treatment of ischemic stoke.

## Materials and Methods

### Animals

Adult male Sprague Dawley (SD) rats weighing 240–260 g were supplied by Changsha Tianqin Biotechnology Co., Ltd. This study was performed under the supervision of Animal Care and Ethics Committee of Wannan Medicial College.

### Experimental Design

100 rats were randomly divided into five groups (20 rats/group) as follows: (1) Sham group: no plug after vessel separation; (2) Ischemia group: plug into vessel for 2 h; (3) Reperfusion group: plug into vessel for 2 h followed by 24 h reperfusion; (4) adenovirus non-load group (Ad-GFP + R): adenovirus non-load inject into rat ventrical followed by Ischemia-Reperfusion; (5) SIK2 overexpression group (Ad-hSIK2+R): 8 days before Ischemia-Reperfusion, adenovirus loaded SIK2 is injected into rat lateral ventricle.

### MCAO and SIK2 Overexpression Model Construction

After 8–10 h of fasting, rats were anesthetized by an intraperitoneal injection of 10% chloral hydrate (0.3–0.5 ml/100 g) and then placed in a supine position. The modified Zea-Longa method (Longa et al., 1989) was referred to for building MCAO model. Rats were placed in the stereotactic device after anesthesia. The rat’s bregma was taken as the origin, and the localization of ventricle followed by AP = −1 mm, VD = 4.5 mm and the right side opening was 2 mm (George and Charles, 2001). Make a mark at the location, then drill a hole with a syringe needle, and inject the virus with a microsyringe finally.

### Hematoxylin–Eosin Staining and 2,3,5-Triphenyltetrazolium Chloride Staining

The obtained brain tissue samples were fixed in 4% neutral-buffered formalin and subsequently embedded in paraffin. Tissue sections (5 μm thick) were stained with HE and analyzed by light microscopy. The fresh brain tissues were frozen for 30 min at −20 °C before being cut into five sections (2 mm thick) along the coronal plane, then these tissues were stained with TTC in 37°C water for 15 min and analyzed by ImageJ.

### Q-PCR Analysis

The obtained brain tissue samples were stored at −80°C condition, then the expression of each protein was measured at the mRNA level for Q-PCR analysis, and lastly the protein level for Western blot was measured. Total RNA was extracted by Trizol. cDNA was prepared by reverse transcription kit. The mixture of cDNA, primer and SYBR Green was subjected to real-time PCR analyses. Primer sequence: SIK2 (5′-TCC​TGC​TTC​CTG​TCA​CTA​T -3′ 3′-TCC​ACG​GCT​TCT​ACC​ATT-5′), HIF-1α (5′-GGA​AAT​GCT​GGC​TCC​CA TT-3′ 3′-CTG​TAA​CTG​GGT​CTG​CTG​GA-5′), mTORC1 (5′-GCTTTGACGC AG GTGCATAG-3′ 3′-TGT​CCC​CAT​AAC​CGG​AGT​AGG-5′), PINK1 (5′-TGATCG AGGAGAACGAGGC-3′ 3′-GCT​TCA​TAC​ACA​GCG​GCA​TT-5′), Parkin (5′-TT TGT​CAG​GTT​CAA​CTC​CAG​C-3′ 3′-CCA​GAG​GCA​TTT​GTT​TCG​TGA-5′).

### Western Blot

Total protein was extracted from tissues by RIPA, and the concentration was measured with BCA Protein Quantitative kit. After denaturing by boiling for 5–10 min, the proteins were loaded, separated by SDS-PAGE gel electrophoresis and transferred onto nitrocellulose filter (NC) membranes. After blocked with 5% skim milk or 5% Bovine albumin (BSA), the NC membranes were incubated with the following antibodies at 4°C overnight: rabbit anti-SIK2 (Thermo Fisher; 1:1000); rabbit anti-HIF-1α (Bioss; 1:1000); rabbit anti-mTOR (Cell Signaling; 1:1000); rabbit anti-p-mTOR (Cell Signaling; 1:1000); rabbit anti-PINK1(Abcam; 1:1000); mouse anti-Parkin (Abcam; 1:1000). After rinsed with Tris-buffered saline with Tween (TBST), these membranes were incubated with goat anti-rabbit or anti-mouse IgG-HRP (Biosharp; 1:10000), and exposed with ECL reagent (Biosharp). β-actin was used as the endogenous control.

### ATP Content in Brain Tissues

The ATP content in the brain tissue was detected by the ultraviolet spectrophotometer using the Rat ATP ELISA kit. Referring to the instructions of the kit, the tissue homogenate was extracted firstly. Then add the standard sample and HRP-Conjugate reagent in the pore plates in turn for the incubation and washing of this mixture. Finally add the chromogenic solution and the stop solution. Check the OD value of each hole on the machine within 15 min after adding the stop solution.

### Transmission Electron Microscopy Observation

The preparation of specimens for TEM observation: Rapidly take the brain tissue out after it being perfused with paraformaldehyde. The surface of the tissue was cleaned with glutaraldehyde solution. A small piece of cortical tissue (with a size of 1 mm^3^) was quickly divided with a sharp blade (within 1 min) and immediately placed in the 2.5% glutaraldehyde solution. Then refrigerate and fix it for more than 2 h at 4°C. Samples were made of resin-embedded blocks, which were cut into 50–70 nm ultrathin sections with an ultramicrotome. The ultrathin sections were double stained by uranium lead and examined with transmission electron microscope operating at 100 kV.

### Statistical Methods

All data were presented as mean ± standard error of the mean (mean ± SEM) and analyzed by the SPSS 13.0 statistical software. The one-way ANOVA was used for the statistical evaluation of these data. The level of significance was set at *p* < 0.05.

## Results

### The Amelioration Effect of SIK2 Overexpression on the Pathological Injury Induced by MCAO

**FIGURE 1:**
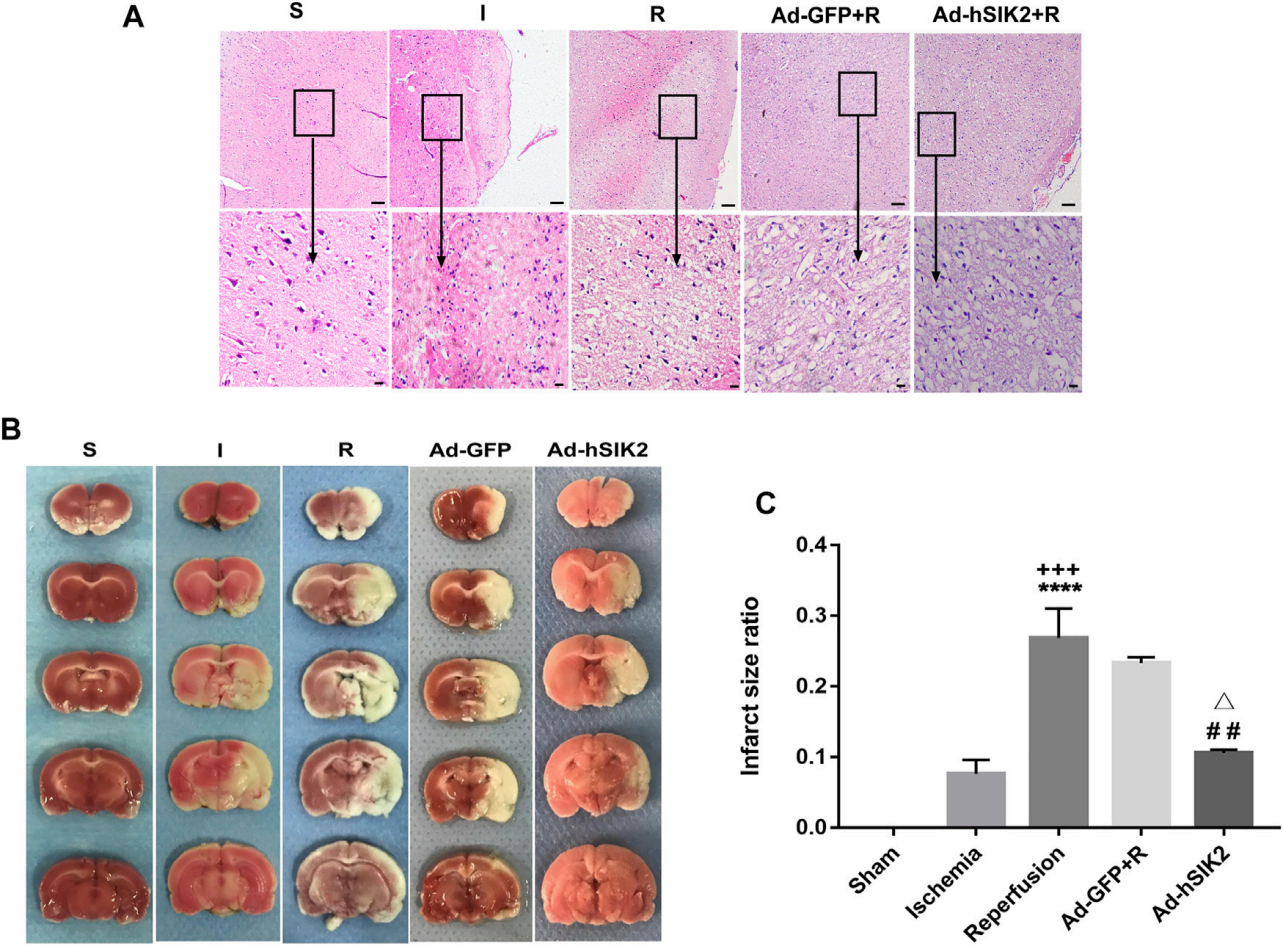
Overexpression of SIK2 can reduce the pathological damage of rat brain tissue. **(A)**.The pathological damage of the brain tissue in each group. Tissues of cortex, the upper row is 10 × 10 low-power lens with a scale of 100μm, the lower row is 10 × 40 high-power lens with a scale of 20 μm. **(B)**.The infarct area of each group. The red area is normal brain tissue, and the white area is the infarcted area tissue. **(C)**.ImageJ analysis of infarct area. Data are the ratio of infarct area to total area as the means ± SEM of *n* = 3 samples. * *vs*. sham group,+ *vs*. ischemia group, # *vs*. reperfusion group, △ *vs*. no-load group.

The pathological changes of brain tissues determined by HE staining in each group were shown in (Figure 1A). In the sham (S) group, the morphology of nerve cells is regular, the cytoplasm is rich, the nucleus is clearly visible, and the vacuolar degeneration is not found. In the ischemia (I) group, the ischemic area is lightly stained, and a small amount of nerve cell liquefaction necrosis can be seen in some areas as sieve-shaped. In the reperfusion (R) group and adeno virus non-load SIK2+reperfusion (Ad-GFP+R) group, a large area of nerve cell liquefaction necrosis can be seen, and the entire visual field under high magnification is covered with vacuolar degeneration. In the SIK2 overexpression + reperfusion (Ad-hSIK2+R) group, part of the cortex is lightly stained, some vacuole-like structures can be seen under high magnification, and the nucleus is clearly visible. TTC staining was shown in Figure 1B. The brain tissue of the S group was normal red with no infarcts. The I group showed a smaller infarct area. The R group and the Ad-GFP + R group showed a larger infarct area, while the infarct size was reduced in the Ad-hSIK2+R group. After analysed by ImageJ software, the difference was statistically significant (Figure 1C).

### SIK2 Overexpression Increasing the Expression of mTOR and HIF-1α

**FIGURE 2:**
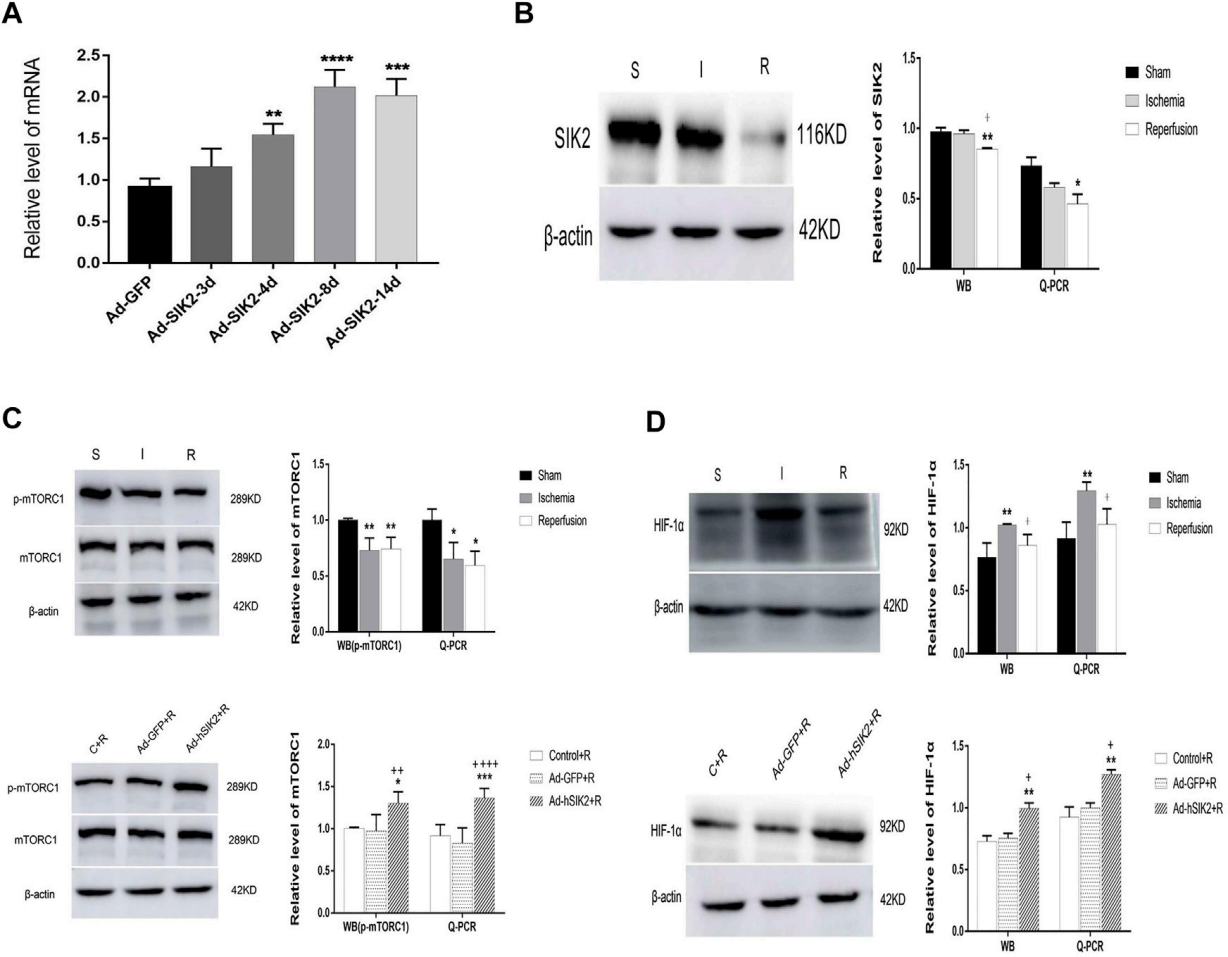
Overexpression of SIK2 can increase the expression of mTOR and HIF-1α. **(A)** The time screening of SIK2 overexpression. On the 8^th^ day of adenovirus injection, the expression level was the highest, which was 2.93 times higher as the normal expression level. * *vs*. Ad-GFP group. **(B)** The protein and mRNA levels of SIK2 in each brain group. **(C)** The protein and mRNA levels of mTORC1 in each group. **(D)** The protein and mRNA levels of HIF-1α in each group. Data are presented as the means ± SEM of *n* = 5 samples. * *vs*. sham group and Control + R group, + *vs*. ischemia group and Ad + GFP group.

Q-PCR was used to detect the level of adenovirus in rat brain tissues at different times after injection. We found that the level of adenovirus was the highest on the 8th day after injection and the mechanical damage of brain tissue was also restored to normal (Figure 2A). Therefore, injecting adenovirus 8 days before MCAO was finally selected to establish the overexpression model. WB and Q-PCR were used to measure the levels of mTORC1 and HIF-1α in each group. Compared with the sham group, the level of SIK2 decreased in the ischemia group and the reperfusion group, and the reperfusion group decreased more significantly than the ischemic group (Figure 2B). Compared with the sham group, the expression of p-mTORC1 in the ischemia group and the reperfusion group both decreased (Figure 2C), while compared with the reperfusion group, the Ad-GFP+R group did not change significantly, and the Ad-hSIK2+R group increased significantly (Figure 2C). Compared with the sham group, the expression of HIF-1α in ischemia group increased (Figure 2D). While compared with the reperfusion group, the Ad-GFP + R group had no significant change and the Ad-hSIK2+R group increased significantly (Figure 2D).

### SIK2 Overexpression Leading to Increased ATP Content

**FIGURE 3:**
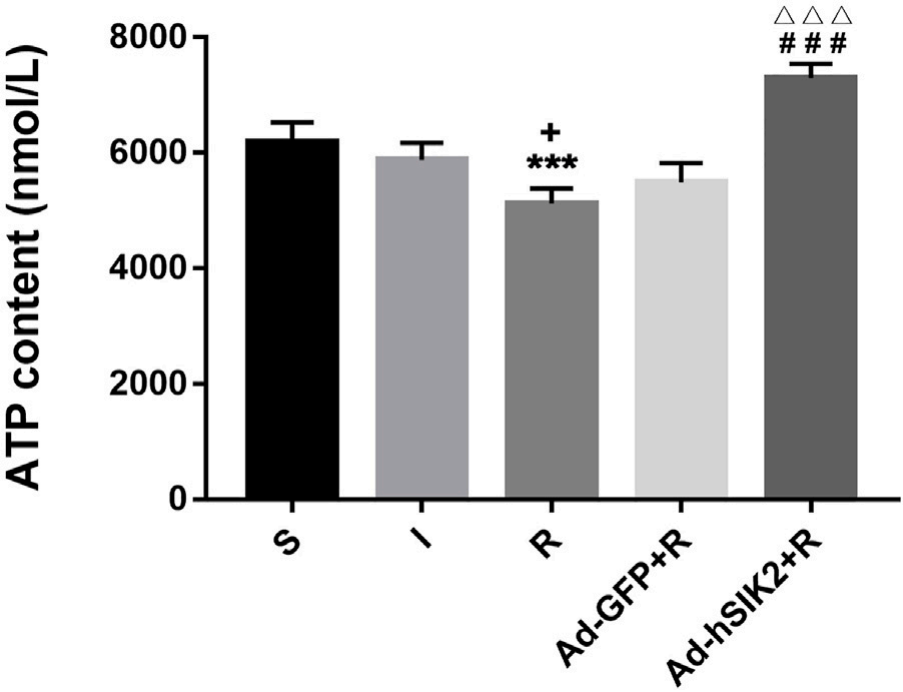
The ATP content of brain tissue increases after SIK2 overexpression. The ATP content in the brain tissue of each group. Data are presented as the means ± SEM of *n* = 5 samples. * *vs*. sham group, + *vs*. ischemia group, # *vs*. reperfusion group, △ *vs*. no-load group.

ELISA kit was used to measure the ATP content in brain tissues of each group. The results showed that compared with the sham (S) group, the ATP contents in ischemia (I) group and the reperfusion (R) group were decreased. And compared with the reperfusion group, ATP content in adeno virus non-load SIK2+reperfusion (Ad-GFP + R) group did not change significantly, while increased significantly in the SIK2 overexpression + reperfusion (Ad-hSIK2+R) group (Figure 3).

### The Stimulation Effect of SIK2 Overexpression on Mitochondrial Autophagy

**FIGURE 4:**
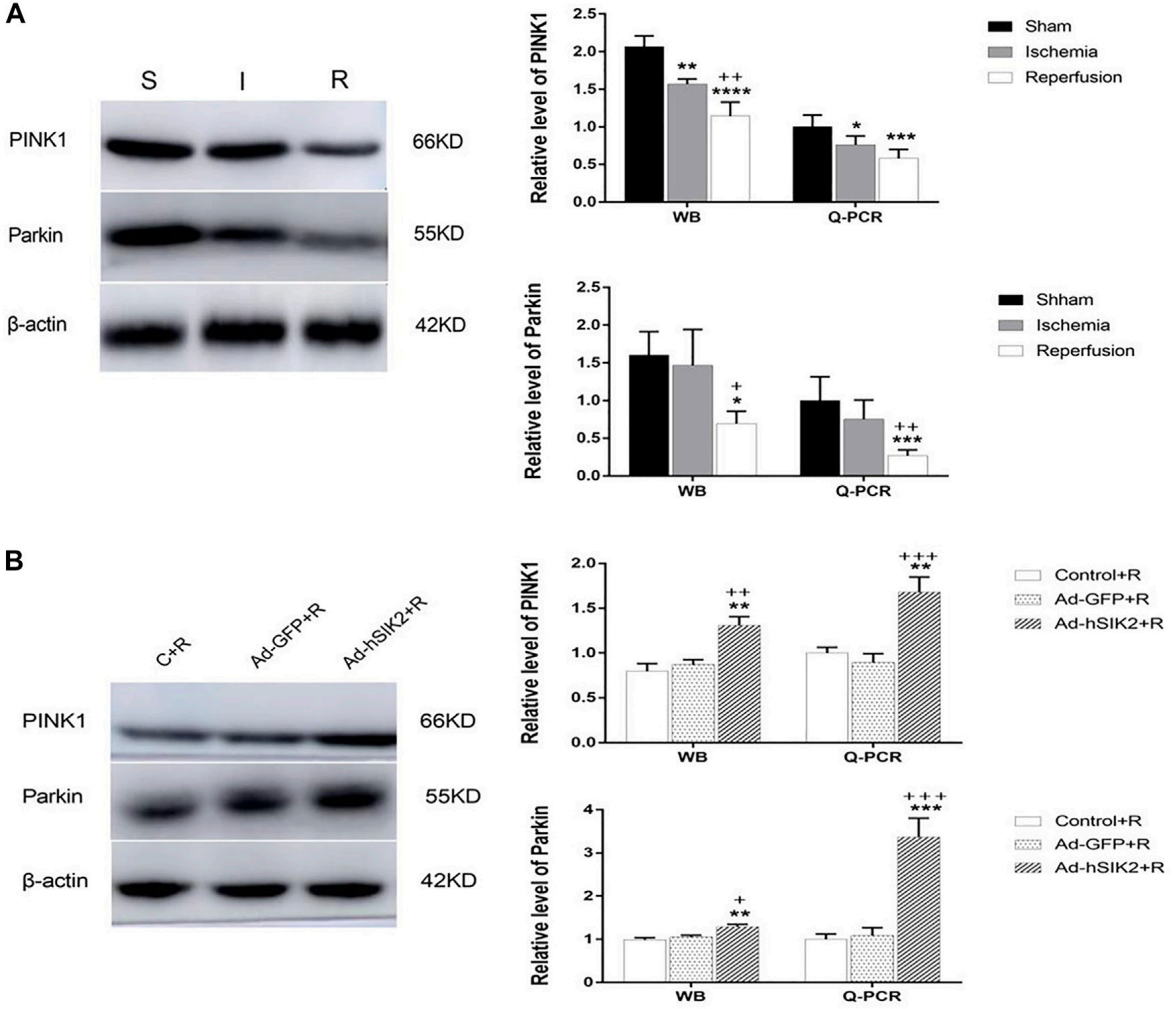
SIK2 overexpression can stimulate mitochondrial autophagy. **(A)** The levels of PINK1 and Parkin in sham, ischemia and reperfusion groups. **(B)** The level of PINK1 and Parkin in reperfusion, non-load and overexpressin groups. Data are presented as the means ± SEM of *n* = 5 samples. * *vs*. sham group and Control + R group, + *vs*. ischemia group and Ad + GFP group.

WB and Q-PCR were used to measure the levels of PINK1 and Parkin in each group. The results showed that compared with the sham group, the level of PINK1 was significantly decreased in ischemia and reperfusion group, and the decrease in reperfusion group was more significant than that in ischemic group (Figure 4A). While compared with the reperfusion group, the Ad-GFP+R group had no significant changes, and the Ad-hSIK2+R group increased significantly (Figure 4A). Then compared with the sham group, significantly decreased level of Parkin was not found in ischemic group, but the reperfusion group was significantly lower than the sham and ischemic group (Figure 4B). While compared with the reperfusion group, significant changes were not found in the Ad-GFP+R group, and the level of the Ad-hSIK2+R group increased significantly (Figure 4B).

### Changes of Mitochondria in Each Group underTransmission Electron Microscope

**FIGURE 5:**
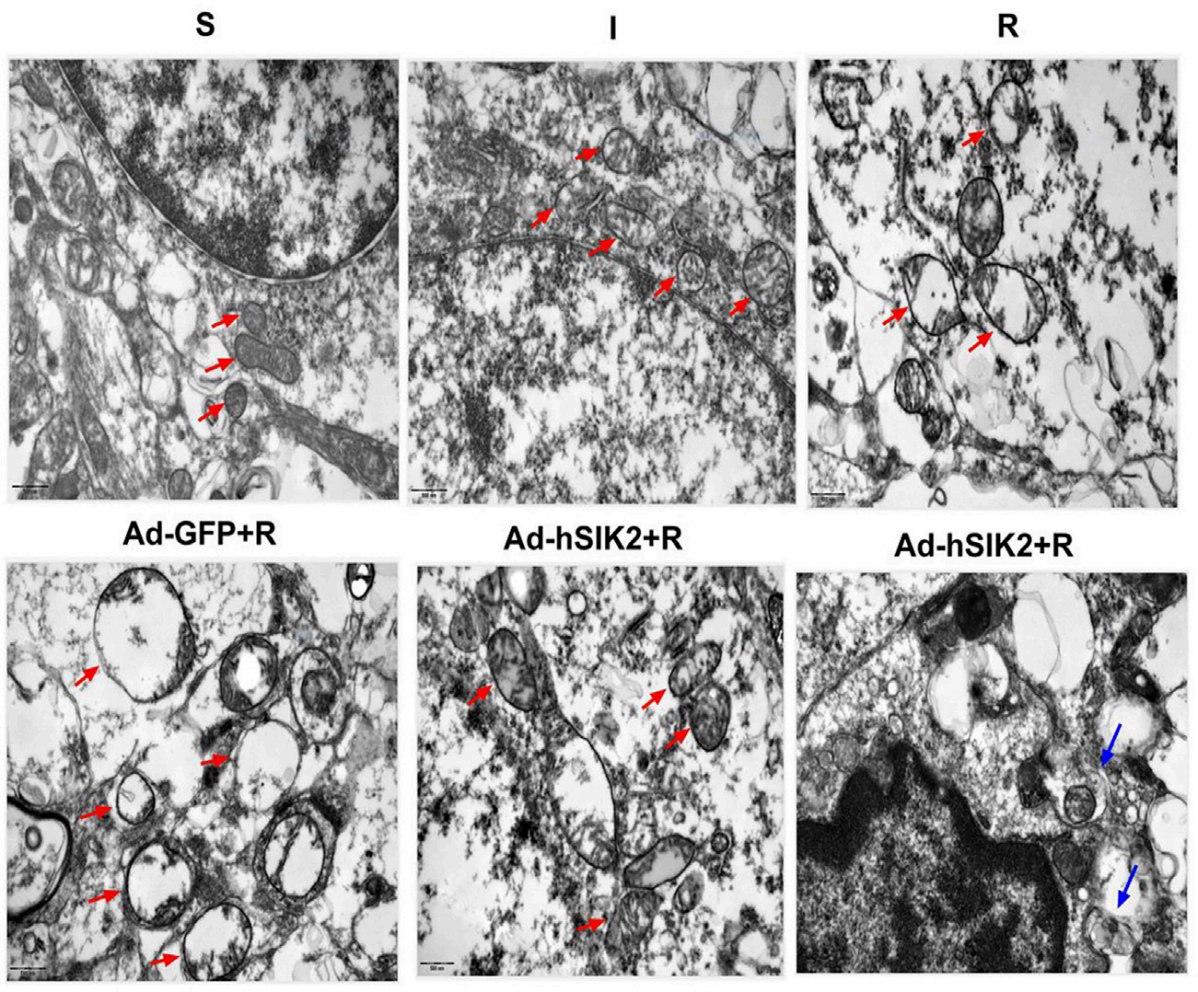
Changes of mitochondria in each group under transmission electron microscope. The red arrow in the figure refers to mitochondria. The morphology and structure of mitochondria in the S group are normal and in the I group are slightly swollen and in the R and Ad-GFP + R groups are highly swollen and vacuolated and in the Ad-hSIK2 + R group are slightly swollen. The blue arrow points to autophagosomes, the organelles wrapped in autophagosome are mitochondria, which is mitochondrial autophagy. The figures above are all 25,000 × microscopically, and the scale is 500 nm.

The ultrastructure of brain tissue was observed by TEM and exhibited in Figure 5. In the sham (S) group, the neure had normal nucleus structure and complete nuclear membrane, with evenly distributed chromatin and other substances in the nucleus. Complete mitochondria could be seen in the cytoplasm, and mitochondrial cristae were clearly visible. In the ischemic (I) group, the neure also had normal nuclear structure and the nuclear membrane was relatively intact, but most of the nuclear material was degraded, the mitochondria in the cytoplasm were slightly swollen, and the mitochondrial cristae was also visible. In the reperfusion (R) and adeno virus non-load SIK2+reperfusion (Ad-GFP+R) group, the nuclear boundaries were blurred and nuclear membrane was broken, while the mitochondria in the cytoplasm were highly vacuolated, and there was no autophagosome. In the SIK2 overexpression+reperfusion (Ad-hSIK2+R) group, nuclear membrane was relatively intact, and the material in nuclear was partially degraded, while the mitochondria were slightly swollen, and mitochondrial cristae and autophagosomes were also visible.

## Discussion

In this study, we confirmed that SIK2 could play a similar role to AMPK in the mTOR pathway, which promoted the phosphorylation expression of mTORC1 and the energy metabolism, induced the occurrence of mitochondrial autophagy, and improved the cerebral ischemia-reperfusion injury. The HE and TTC staining clarified that the overexpression of SIK2 and the neuronal and mitochondrial degeneration were alleviated; meanwhile, the brain tissue infarct size was decreased, the levels of mTORC1 and HIF-1α were increased as same as the PINK1 and Parkin, and so were the ATP content. So we conclude that SIK2 can improve the mitochondrial autophagy restriction which is induced by cerebral ischemia-reperfusion to promote the occurrence of energy metabolism through the mTOR pathway in rats.

PINK1 is a protein encoded by the human chromosome 10 homologous deletion phosphatase-tensin gene-induced kalubizyme 1, and its structure contains mitochondrial targeting sequences. Parkin is an E3 ubiquitin ligase, and its imbalance is related to Parkinson’s disease and substantia nigra neuron loss (Youle and Narendra, 2011). In normal cells, PINK1 is rapidly degraded by proteolysis and maintains a low level. While in damaged mitochondria, PINK1 hydrolysis is inhibited and it promotes the recruitment of Parkin to the mitochondria, which inhibits mitochondrial fusion through ubiquitination of mitochondrial fusion proteins to activate mitochondrial autophagy (Tanaka et al., 2010). The autophagy-lysosome process can regulate the degradation and reuse of damaged organelles in the cell, and the PI3K/Akt/mTOR pathway is a key regulator which induces autophagy and regulates autophagosome formation (Liu et al., 2019). A study has shown that the electroacupuncture reduces the neuronal damage during cerebral ischemia-reperfusion by improving the clearance of damaged mitochondria through mitochondrial autophagy. This process also activates the PI3K/Akt/mTOR signaling pathway (Wang et al., 2019). Our results showed that SIK2 overexpression increased the levels of mTORC1, PINK1 and Parkin, indicating that mitochondrial autophagy could promote the removal of damaged mitochondria. Bcl2/adenovirus E1b interacting protein 3 (BNIP3) is a mitochondrial outer membrane protein, and it is also necessary for mitochondrial autophagy clearance (Ney, 2015; Fu et al., 2018). Studies have shown that increased level of HIF-1α can activate BNIP3. And up-regulating the level of HIF-1α and BNIP3 may promote autophagy of myocardial cell after ischemia-reperfusion injury and H9C2 cells induced by OGD/R. In addition, down-regulating the level of HIF-1α or BNIP3-siRNA can reduce the autophagy of H9C2 cells under OGD/R. Therefore, the HIF-1α/BNIP3 signaling pathway can protect the myocardial ischemia-reperfusion injury by inducing autophagy (Zhang et al., 2019). Our results showed that SIK2 overexpression increased the level of HIF-1α. HIF-1α can promote glycolysis under hypoxic conditions and increase the production of ATP, which indicates that HIF-1α may also promote the production of ATP by regulating mitochondrial autophagy.

Various studies have confirmed that SIK2 participates in cell growth and metabolism, which is closely related to the chemotherapy of ovarian cancer (Ahmed et al., 2010). The experiments we completed before have confirmed that SIK2 had impact on the brain tissue damage after cerebral ischemia-reperfusion in rats by regulating angiogenesis. Furthermore, SIK2 can participate in cell energy metabolism. Therefore, this study aims to explore whether SIK2 can also regulate neuronal damage from energy metabolism to protect brain tissues. Besides, it is believed that further investigations and experiments are needed to explore the broad and far-reaching significance of energy metabolism.

The detection of autophagy is more complicated and diverse, and TEM is the gold standard for detecting mitochondrial autophagy (Prachar, 2017). For cell samples, co-localization of lysosomes and mitochondria can also be used for comprehensive evaluation except TEM (Park et al., 2015). However, TEM is the most effective method for tissue specimens at present. Therefore, the process of obtaining tissue specimens is very important, which is highly possible affect the results. This point needs further improvement.

## Conclussion

It is concluded that SIK2 can be used as a positive regulator of cerebral ischemia-reperfusion injury to reduce the brain tissue damage. This result may provide a new target for the ischemic stroke treatment in clinical.

## Data Availability

The original contributions presented in the study are included in the article/Supplementary Material, further inquiries can be directed to the corresponding authors.
